# Critical current modulation induced by an electric field in superconducting tungsten-carbon nanowires

**DOI:** 10.1038/s41598-021-97075-z

**Published:** 2021-09-06

**Authors:** Pablo Orús, Vladimir M. Fomin, José María De Teresa, Rosa Córdoba

**Affiliations:** 1 Instituto de Nanociencia y Materiales de Aragón (INMA), CSIC-Universidad de Zaragoza, 50009 Zaragoza, Spain; 2 Departamento de Física de la Materia Condensada, Facultad de Ciencias, Universidad de Zaragoza, 50009 Zaragoza, Spain; 3 Institute for Integrative Nanosciences (IIN), Leibniz Institute for Solid State and Material Research (IFW) Dresden, Helmholtzstraße 20, 01069 Dresden, Germany; 4 Laboratory of Physics and Engineering of Nanomaterials, Department of Theoretical Physics, Moldova State University, Strada A. Mateevici 60, 2009 Chişinău, Republic of Moldova; 5 Institute of Engineering Physics for Biomedicine, National Research Nuclear University MEPhI, Kashirskoe shosse 31, Moscow, 115409 Russia; 6 Laboratorio de Microscopías Avanzadas (LMA), University of Zaragoza, 50018 Zaragoza, Spain; 7 Instituto de Ciencia Molecular (ICMol), Universitat de València, 46980 Paterna, Spain

**Keywords:** Superconducting properties and materials, Superconducting devices

## Abstract

The critical current of a superconducting nanostructure can be suppressed by applying an electric field in its vicinity. This phenomenon is investigated throughout the fabrication and electrical characterization of superconducting tungsten-carbon (W-C) nanostructures grown by Ga$^+$ focused ion beam induced deposition (FIBID). In a 45 nm-wide, 2.7 $\upmu$m-long W-C nanowire, an increasing side-gate voltage is found to progressively reduce the critical current of the device, down to a full suppression of the superconducting state below its critical temperature. This modulation is accounted for by the squeezing of the superconducting current by the electric field within a theoretical model based on the Ginzburg–Landau theory, in agreement with experimental data. Compared to electron beam lithography or sputtering, the single-step FIBID approach provides with enhanced patterning flexibility and yields nanodevices with figures of merit comparable to those retrieved in other superconducting materials, including Ti, Nb, and Al. Exhibiting a higher critical temperature than most of other superconductors, in which this phenomenon has been observed, as well as a reduced critical value of the gate voltage required to fully suppress superconductivity, W-C deposits are strong candidates for the fabrication of nanodevices based on the electric field-induced superconductivity modulation.

## Introduction

In the semiconductor industry, the term *field effect* is used to define the modulation of the charge carrier density that takes place in the active channel of a semiconductor when an electric field is externally applied^1^. This electric field-induced resistivity control is at the core of the operation of field-effect transistors (FETs). FETs are employed as active electronic components that exploit the field effect for electrical signal switching and amplification^2^. Particularly, metal oxide semiconductor FETs (MOSFETs) fabricated on integrated circuits represent the most extensively used building block in that industry for the fabrication of a wide range of electronic-based devices thanks to their simplicity and affordability^3^.

The field effect is virtually non-existent in metallic materials, since the large density of charge carriers present in a metal promptly results in the screening of externally applied electric fields over very short distances. However, recent calculations indicate that such a field is able to extend within a superconducting material over at least the value of its coherence length^4^, and the study of the phenomenon in superconducting materials has gained interest in recent years due to its potential application in superconducting electronics^5^.

Central to this interest is the fact that an increasing electric field applied in close proximity to a superconducting bridge has been recently found to progressively suppress the critical current of superconducting channels. This electric field-induced quenching of the superconducting state has been observed in different superconducting materials, mostly purely metallic: in titanium transistor-like devices, both in-plane^6^ and suspended^7^; in aluminium nanodevices with a single back-gate^8^ or side-gate^9^, deducing from the latter a weak coupling between the electric field and an external magnetic field; in vanadium Dayem nano-bridges, operated as electrical rectifiers^10^; and in niobium gate-controlled transistors, also exploited as half-wave rectifiers^11^. Uniquely, and contrarily to other works, an *enhancement* of the critical current with increasing gate voltage has also been observed in niobium nitride thin micro- and nano-bridges, being ascribed to changes in the superconducting vortex surface barrier^12^.

The microscopical justification for the occurrence of this effect, which cannot be justified in the Barden–Cooper–Schrieffer (BCS) framework, is not fully accounted for at the present time and is the subject of an unsettled debate^13–15^, with separate intrinsic and extrinsic effects potentially contributing in various geometries and in different superconducting materials. Ritter et al.^16^ ascribe the critical current quenching observed in titanium nitride nanowires to the injection of energetic electrons from the gate electrodes to the superconductor, which trigger the formation of a large number of quasiparticles that drive the nanowire back to the normal state. Alegria et al.^17^ and Golokolenov et al.^18^ support similar arguments to account for the behavior observed in electron tunneling spectroscopy experiments in titanium nanowires and in a vanadium waveguide resonator, respectively. On the other hand, Mercaldo et al.^19^ propose a theoretical model based on electric-field induced spin-orbit polarization at the surface, capable of modulating the phase and amplitude of the superconducting order parameter. Similarly, Chirolli et al.^20^ present a model for crystalline superconductors, in which the effect is accounted for by a local modification of the density of states of the material induced through Rashba-like surface effects. Solinas et al.^21^ describe a Sauter–Schwinger effect in BCS superconductors to account for the phenomenon.

The empirical observation of the superconducting current quenching proves a highly promising path towards the fabrication of superconducting FET (S-FET) nanodevices, which can simultaneously benefit from the non-dissipating regime characteristic to superconducting materials, and from the electric switching capability of devices controlled by the application of a gate voltage^22,23^. Additional prospective applications include the exploitation of the effect in spin-filter Josephson junctions^24^, logic gates^25^, and single-photon detectors^26^.

The precise nanofabrication of such devices can be achieved by means of the *focused ion beam induced deposition* (FIBID) procedure. FIBID is a direct-write nanopatterning technique that makes use of a focused beam of energetic Ga$^+$ ions (FIB) to locally induce the decomposition of a gaseous precursor material previously adsorbed on the surface of the substrate. With a spot size of 5 nm, as the beam scans the surface of the substrate, the gaseous monolayer of precursor is decomposed at and near the irradiated area following the pattern traced by the beam, resulting in the growth of a pattern-shaped nanodeposit^27,28^. A prime example of the capabilities of this technique is the recently reported fabrication of NbC nanodevices for vortex dynamic investigation via FIBID of a Nb-based gaseous precursor^29^.

FIBID of the W(CO)$_6$ precursor results in the growth of a W-C based material with an atomic content of W of 40-50%, a nanocrystallite-based microstructure, and a room-temperature resistivity (ρ300 K) of 200–300 $\upmu \Omega$ cm. Paramountly, the material is type-II superconductor, with a critical temperature ($T_c$) around 4.75 K, an upper critical magnetic field of 9.5 T, a critical current density of 0.01-0.10 MA/cm$^2$, and values for the coherence length and penetration depth of 6.25 nm and 850 nm, respectively^30–35^. Superconductivity has also been found to occur in the material yielded when the decomposition of W(CO)$_6$ is induced by a focused electron beam instead, which has been exploited for the fabrication of Josephson junctions^36^. Remarkably, and contrary to most of the aforementioned superconducting materials, in which this phenomenon has been investigated, Ga$^+$ FIBID W-C is not fully metallic, nor it is a highly crystalline, yet it allows for the occurrence of the effect, as it will be presented in the following sections. The novel exploitation the Ga$^+$ FIBID technique for the direct nanopatterning of W-C S-FET devices further settles it as a solid technique for the fabrication of superconducting components in advanced nanodevices.

The patterning flexibility and the single-step nature of the FIBID procedure make for highly convenient clean growth and handling of a superconducting material. As such, Ga$^+$ FIBID of W-C is a strong approach for the further investigation of electric field-induced superconductivity modulation. The experimental observation of such a phenomenon in Ga$^+$ FIBID W-C nanowires is presented here. A full suppression of the superconducting state below the critical temperature of the material is achieved at a remarkably low side-gate voltage of 3 V. A theoretical model based on the Ginzburg–Landau (GL) theory is provided, which appropriately reproduces the experiments. The transconductance of the nanodevice is comparable by value to that retrieved in the aforementioned works in other superconducting materials, further fueling the potential of FIBID of W(CO)$_6$ as a solid technique for the fabrication of S-FET devices.

## Methods

Si/SiO$_2$ pieces were used as substrates. A supporting Cr/Au structure consisting of current/voltage and gate electrodes was defined by electron beam lithography (EBL) in a Raith Group *PIONEER Two* EBL/Scanning Electron Microscope (SEM) instrument, and grown in an electron-beam evaporator, eliminating the EBL resist by liftoff.

The two gate contacts were initially patterned as a continuous, 45 nm-thick film. A 200nm-wide, 2.7 $\upmu$m-long gap was milled between the two selectively removing material using the Ga$^+$ FIB^37^ exposing the underlying Si/SiO$_2$ substrate. The milling was carried out using the same operating parameters employed in growth (without the injection of the precursor material) and in the same instrument, both described below.

FIBID and scanning electron microscopy (SEM) imaging of the samples were carried out in a commercial Thermo Fisher *Helios 600 NanoLab* FIB/SEM microscope, equipped with a Ga$^+$ column with a liquid metal ion source. In the FIBID procedure, an acceleration voltage of 30 kV and an ion beam current of 1.8 pA were used. The process chamber had a base pressure of $10^{-6}$ mbar, which was raised to $10^{-5}$ mbar during the injection of the gaseous W(CO)$_6$ precursor. During growth, the nozzle used to deliver the precursor was positioned 50 $\upmu$m away from the irradiation point in the vertical direction, and 100 $\upmu$m away in the in-plane direction.

**Figure 1:**
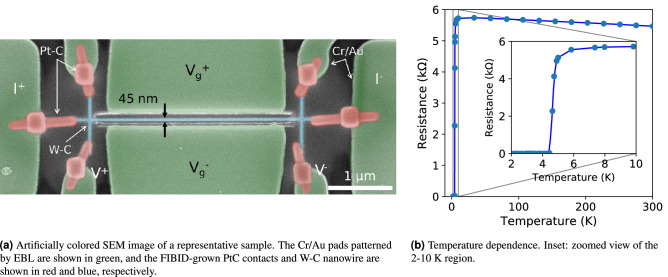
SEM image and superconducting transition of a nanodevice. In (**b**), solid lines are a guide for the eye.

The deposits were patterned as straight, narrow nanowires with two (equally wide) perpendicular leads crossing at each end, with length between leads of 3.3 $\upmu$m, width of 45 nm (nominally 20 nm), and a nominal thickness of 30 nm (Fig. 1a). The used values of volume per dose, dwell time, and overlap were to $8.2\cdot 10^{-2}$ $\upmu$m$^3$/nC, 500 $\upmu$s, and 50%, respectively. The total deposition time for each deposit was 15 seconds. Growing the deposit in the 200 nm-wide gap yielded in a nanowire-electrode separation of around 75 nm at each side.

Lastly, the ends of the nanowire were joined to the metallic pads by growing supporting electrical contacts via FIBID of the (CH$_3$)$_3$(CH$_3$CpPt) precursor, known to present a metallic content high enough to be used for this purpose^38,39^. They were grown as 150 nm-wide, 100 nm-thick leads with a supporting 300$\times$300 nm, 100 nm-thick square grown at the joining point with the pads. In this FIBID procedure, the ion beam current, acceleration voltage, dwell time, and overlap were set to 1.8 pA, 30 kV, 200 ns, and 0%, respectively. The volume per dose in this growth was 0.5 $\upmu$m$^3$/nC.

The device was designed in this manner for the driving current to be injected along the long axis of the nanowire while the voltage drop was measured in the 3.3 $\upmu$m-long channel between the leads, in a four probe measurement fashion. The gate voltage was applied using the two large pads located in close proximity to this channel.

The low-temperature electrical characterization of the samples was performed in a commercial Quantum Design *Physical Property Measurement System* instrument. Measurements were taken down to 2 K. Electrical contact between the instrument and the sample was achieved by ultrasonic wire-bonding of aluminum wires of the EBL-patterned metallic contacts to the instrument sampleholder.

## Results and discussion

The experimental results strengthen the applicability of Ga$^+$ FIBID of W(CO)$_6$ for the fabrication of S-FET nanodevices. Superconductivity in W-C nanowires patterned using this technique is experimentally found to be progressively suppressed with increasing values of gate voltage, up to a full transition to the normal state for a gate voltage of around 3 V at 2 K.

### Experimental results

The nanowires exhibited an estimated room-temperature resistivity of 230 $\upmu \Omega \cdot$cm and a temperature-induced transition to the superconducting state in the vicinity of $T_c=4.75$ K (defining $T_c$ as the temperature at which the resistance takes half its value in the normal state, at 10 K) (Fig. 1a). Contrary to Al, Ti, and Nb, and similarly to the NbN material, Ga$^+$ FIBID W-C presents a non-metallic response above its critical temperature, with a residual-resistivity ratio at 10 K ($\rho _{\text {300 K}}/\rho _{\text {10 K}}$) of 0.96.

**Figure 2:**
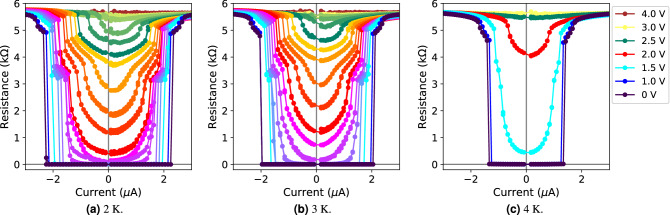
Electric field-induced critical current modulation at 2, 3, and 4 K under gate voltages from 0 to 4 V. Solid lines are a guide for the eye. In (**a**) and (**b**) all unlabeled curves (between 1.5 and 3.0 V) are 0.1 V apart.

The modulation of the critical current induced by the electric field was assessed by measuring current–resistance curves of the nanowire below the critical temperature while sequentially increasing the voltage difference between the gate electrodes, thus obtaining the resistance as a function of the driving current (Fig. 2). To account for possible hysteresis effects, each measurement consisted of a complete cycle, increasing the driving current from 0 to ± 3 $\upmu$A, and then decreasing it again down to zero.

The critical current of the device (here defined as the value of the driving current at which the resistance of the device reaches 90% of its non-superconducting, normal value) monotonically decreased with increasing gate voltage. In the absence of gate voltage, at 2 K a critical current density of 0.24 MA/cm$^2$ is detected. The depairing current density^40,41^ takes an estimated value of 2.37 MA/cm$^2$. The obtained values of critical current density are comparable to those obtained in different devices of W-C grown via FIBID, with both 2D^35^ and 3D^42^ Ga$^+$ and He$^+$ W-C FIBID nanostructures, respectively. A comparable critical current density of 0.18 MA/cm$^2$ is reported in similar Ti nanodevices^43^.

At 2 K, a complete suppression of the superconducting state for applied gate voltages of around 3 V (Fig. 2a) was observed. The response of the device is asymmetric with respect to the sign of the current: for positive values of the gate voltage, the onset of the modulation is favored at negative values of the driving current. This effect cannot be fully accounted for at the present time, although it is not unreasonable for it to be caused by leakage current effects flowing to and from the gate contacts^15^.

No hysteresis was detected in the devices, indicating that no significant thermal heating effects take place in the nanowires at the experiment conditions. Similar behavior was observed at temperatures of 3 and 4 K (Fig. 2b,c, respectively).

Some resistance–current curves exhibited a transition to the normal state in the form of two independent steps, pointing towards the local appearance of non-superconducting areas in the superconducting channel that yield a non-zero resistance as the current flows through. These localized normal regions appear by the electric field-induced squeezing of the superconducting current, which gains relevance as the gate voltage and the temperature increase. The squeezing eventually leads to the whole nanowire being forced into the normal state below its critical temperature (further discussion on this matter follows in the next section).

**Figure 3:**
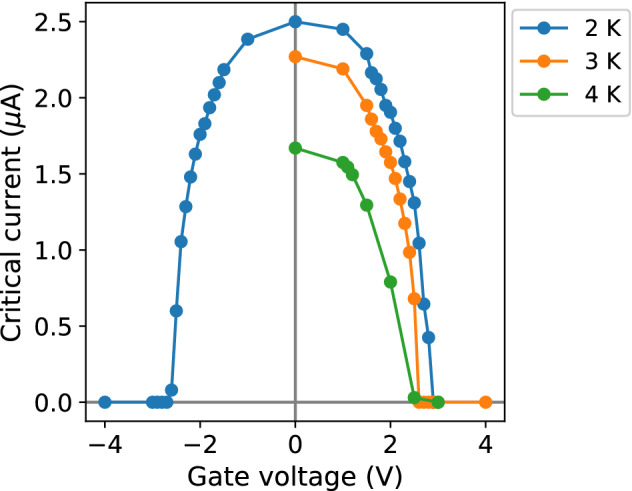
Critical current as a function of the gate voltage. Solid lines are a guide for the eye.

Similarly, finite resistance values were detected at very low values of driving current for gate voltage values above 2 V at 2 K. At 3 and 4 K, as the critical current of the device decreased with increasing temperature, the extent of the electric field-induced modulation was also shifted towards lower values of the current (Fig. 3).

Between gate voltages of 2 and 3 V, at very low values of driving current the nanowire is already in a resistive state (yet with an absolute value of resistance lower than that in the normal state), also in agreement with the proposed interpretation in terms of the squeezing effect.

### Theoretical model

De Simoni et al.^43^ proposed an ad hoc theoretical model based on the GL theory to describe the experimentally observed modulation of the critical current in titanium nano-bridges as a function of temperature and gate voltage. A description which is presented here, is also built in the GL framework, providing with added robustness by including the boundary conditions.

In a superconducting wire affected by an external electric field, the superconducting current experiences a squeezing effect by the electric field perpendicular to the wire. Therefore, to develop a *minimal model* of the critical current in a superconducting wire, the two key conceptual ingredients are the superconducting current flowing along the wire, and the compression of the superconducting state across the wire.

The proposed model of the critical current in a superconducting wire of length *L* (along the *x*-axis) and a rectangle-shaped cross-section sized $w_y\times w_z$ (along the *y*- and *z*-axes, respectively), is based on the GL free energy functional *F*^40^:

1 $$\begin{aligned} F = \xi ^2\vert \nabla \psi \vert ^2-\vert \psi \vert ^2+\frac{1}{2\psi _0^2}\vert \psi \vert ^4+F_n, \end{aligned}$$

where $F_n$ is the free energy of the normal state and other fields, $\xi$ is the coherence length, and $\psi$ is the order parameter, with $\psi _0$ being the value it takes infinitely deep in the bulk of the superconductor. It is supplemented with the boundary conditions on the edges^40^, both with the surrounding insulator:

2 $$\begin{aligned} \nabla _y\psi \vert _{y=\pm w_y/2}=0,\qquad \nabla _z\psi \vert _{z=\pm w_z/2}=0, \end{aligned}$$

and with the contacts. As a simplification, the latter are selected in the form of equality of the superconducting current *j*^40^ and the transport current $j_{tr}$ densities at the contacts:

3 $$\begin{aligned} j=\frac{e^*}{m^*}\vert \psi \vert ^2\hslash \nabla _x({\text {Arg}}\psi )\vert _{{\text {contact}}}\equiv j_{tr}, \end{aligned}$$

where $e^*$ and $m^*$ are the effective charge and mass, respectively.

The basic conceptual ingredients of the model are as follows: (i) The order parameter in the superconducting regions carrying the superconducting current along the *x*-axis can be selected in the form of a plane wave,

4 $$\begin{aligned} \psi =e^{ikx}\psi _1(y,z), \end{aligned}$$

with the parameter *k* determining the superconducting current (shown below). (ii) For the effect of the external electric field, the order parameter can be used in the form of the wave function of a ground state of a harmonic oscillator normalized within the wire^43^:

$$\begin{aligned} \psi _{\sigma _y}(y)= & {} \sqrt{w_y}\frac{\exp \left( -\frac{y^2}{4\sigma ^2_y}\right) }{\sqrt{\sqrt{2\pi }\sigma _y}},\\ \psi _{\sigma _z}(z)= & {} \sqrt{w_z}\frac{\exp \left( -\frac{z^2}{4\sigma ^2_z}\right) }{\sqrt{\sqrt{2\pi }\sigma _z}}, \end{aligned}$$

where the dispersion values $\sigma _y$ and $\sigma _z$ (along the *y*- and *z*-axes, respectively) are inversely proportional to the corresponding components of the electric field^43^: $1/\sigma _y=E_y/V_y$ and $1/\sigma _z=E_z/V_z$. A significant compression of the area of superconductivity towards the center of the cross-section implies $\sigma _y\ll w_y$ and $\sigma _z\ll w_z$, leading to an exponentially strong decay towards the edges, so that the boundary conditions (Eq. 3) are approximately satisfied with high accuracy.

Thus, the order parameter can be selected in the form of Eq. (4) with the Ansatz $\psi _1(y,z)=\psi _2\psi _{\sigma _y}(y)\psi _{\sigma _z}(z)$ to retrieve the superconducting current as

5 $$\begin{aligned} I(k)=\int ^{w_y/2}_{-w_y/2}\int ^{w_z/2}_{-w_z/2}jdydz=\frac{\psi ^2_2\hslash e^*}{m^*}w_yw_zk. \end{aligned}$$

A variation of the functional *F* (Eq. 1) with respect to $\psi _1$ yields the GL equation:

6 $$\begin{aligned} \xi ^2\left[ k^2+\left( \frac{y}{2\sigma ^2_y}\right) ^2+\left( \frac{z}{2\sigma ^2_z}\right) ^2\right] \psi _1-\psi _1+\frac{1}{\psi ^2_0}\psi ^3_1=0. \end{aligned}$$

Averaging Eq. (6) over the cross-section of the wire yields $\psi _2$ and subsequently the superconducting current *I* (Eq. 5) as a cubic function of *k*:

7 $$\begin{aligned} I(k)=\frac{\psi ^2_0\hslash e^*}{m^*}w_yw_z\left( k\left[ 1-\frac{\xi ^2}{48}\left( \frac{w^2}{\sigma ^4_y}+\frac{v^2}{\sigma ^4_z}\right) \right] -\xi ^2k^3\right) . \end{aligned}$$

A condition for the current to be maximal is a vanishing derivative of *I*(*k*), which results in:

$$\begin{aligned} k^2=\frac{1}{3\xi ^2}\left[ 1-\frac{\xi ^2}{48}\left( \frac{w^2_y}{\sigma ^4_y}+\frac{w^2_z}{\sigma ^4_z}\right) \right] , \end{aligned}$$

and the critical current follows as

8 $$\begin{aligned} I_c\equiv {\text {max}}\,I(k)=\frac{2\psi _0^2\hslash e^*}{3\sqrt{3}m^*\xi }w_yw_z\left[ 1-\frac{\xi ^2}{48}\left( \frac{w^2_y}{\sigma ^4_y}+\frac{w^2_z}{\sigma ^4_z}\right) \right] . \end{aligned}$$

Using the explicit dependence on temperature,

$$\begin{aligned} \psi ^2_0(T)=\psi ^2_0(0)\left( 1-\frac{T}{T_c}\right) ,\qquad \xi (T)=\xi (0)\left( 1-\frac{T}{T_c}\right) ^{-1/2}, \end{aligned}$$

we arrive at

9 $$\begin{aligned} I_c=\frac{2\psi _0^2(0)\hslash e^*}{3\sqrt{3}m^*\xi (0)}w_yw_z\left( 1-\frac{T}{T_c}\right) ^{3/2}\left[ 1-\left( 1-\frac{T}{T_c}\right) ^{-1}\left( \frac{w^2_y}{\sigma ^4_y}+\frac{w^2_z}{\sigma ^4_z}\right) \right] ^{3/2}. \end{aligned}$$

If the electric field is applied in the *y*-direction, Eq. (9) results in:

10 $$\begin{aligned} I_c(T,E)=I_c(0,0)\left( 1-\frac{T}{T_c}\right) ^{3/2}\left[ 1-\left( 1-\frac{T}{T_c}\right) ^{-1}\left( \frac{E}{E_c}\right) ^4\right] ^{3/2}, \end{aligned}$$

where $E_c$ (and correspondingly, $V_g^c=E_cw_y$) represents the critical value of the electric field (gate voltage), at which superconductivity is fully suppressed at zero temperature, and

11 $$\begin{aligned} I_c(0,0)=\frac{2\psi _0^2(0)\hslash e^*}{3\sqrt{3}m^*\xi (0)}w_yw_z, \qquad \frac{1}{E_c^4}=\frac{\xi ^2(0)w^2_y}{48V^4_y}. \end{aligned}$$

**Figure 4:**
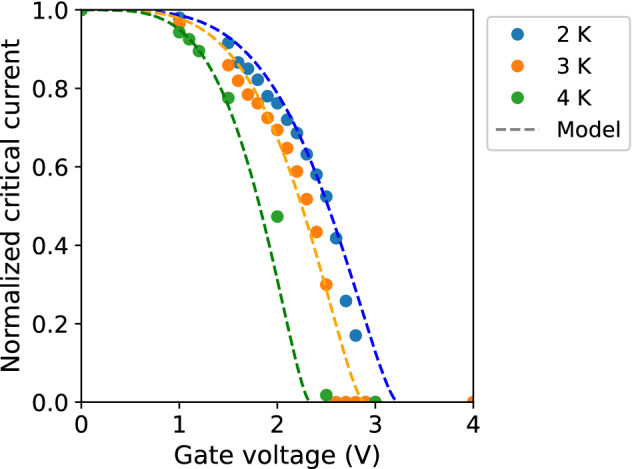
Normalized critical current–gate voltage characteristic. Dots indicate the experimental data at 2, 3, and 4 K. Dashed lines represent the predicted characteristics at the corresponding temperatures (Eq. 10) with $V_g^c=3.7$ V.

The critical current as a function of the gate voltage (Eq. 10) is in good agreement with the experimental data (Fig. 4). Importantly, the critical value of the gate voltage, at which superconductivity is fully suppressed, decreases with increasing temperature.

### Comparison to other materials

**Figure 5:**
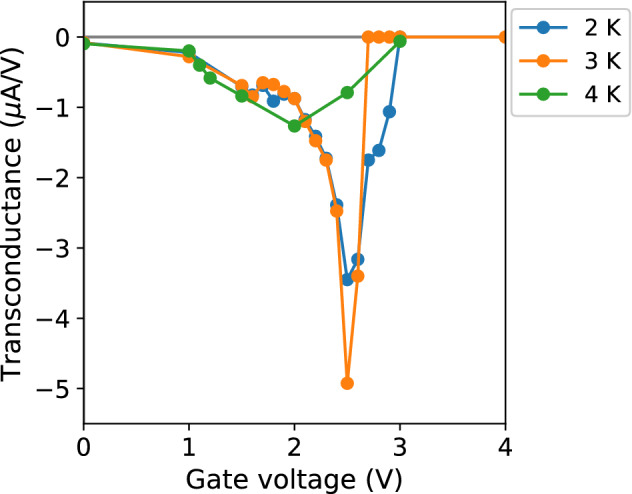
Transconductance. Solid lines are a guide for the eye.

The performance of S-FET devices is typically assessed by means of the *transconductance*, a figure of merit which quantifies the variation in the current with the applied gate voltage, and is calculated here as the numerical derivative of the $I_c(V_g)$ experimental curves, $g_m=dI_c/dV_g$. The absolute values and temperature trend of the transconductance in the W-C nanodevices (Fig. 5) are both comparable to those of other S-FET devices made of other superconducting materials, with the notable difference of a reduced value of critical gate voltage (Table 1).

**Table 1:** Maximum values of the relevant parameters in S-FET nanodevices. Quantities in parentheses indicate the reduced temperature $T/T_c$ at which the corresponding parameter was retrieved.

	${\hbox {T}}_{\mathrm{c}}$ (K)	${\hbox {V}}_{\mathrm{g}}^{\mathrm{c}}$ (V)	${\hbox {g}}_{\mathrm{m}}$ ($\upmu$A/V)
W-C	4.75	3 *(0.421)*	5 *(0.631)*
Ti^6^	0.54	32/8 *(0.093)*	15 *(0.278)*
V^8^	5	13 *(0.675)*	150 *(0.575)*
Al^9^	0.60	25 *(0.083)*	–
Nb^11^	7.90	> 40 ($\gtrsim$* 0.253*)	1.6 *(0.004)*

The response of W-C nanostructures to the electric field does occur in the reduced temperature interval $T/T_c=(0.421,0.842)$ (between 2 and 4 K), and most importantly, a full suppression of the superconducting state is achieved at the low voltage of $V_g^c\sim 3$ V at $T/T_c=0.421$ (2 K), one order of magnitude below the majority of the reported values for other superconductor materials, with the lowest figure (observed in Ti nanostructures) still exceeding by 2.6 times the value retrieved in W-C. The associated values of reduced temperature at which the maximum values of transconductance are detected are on par with those reported in vanadium nanodevices^8^, and above most of the other studied materials. The absolute value of $T_c$ in Ga$^+$ FIBID provides a nanodevice operating temperature range wider than most of the other reported materials, surpassed only by Nb and competitive with V.

Moreover, FIBID is a direct nanopatterning technique, requiring a single step to grow nanostructures patterned at will, offering great flexibility in pattern design and shape fidelity without requiring the use of resists, solvents, etc. that might compromise the final quality of the nanostructures.

## Conclusions

The modulation of the critical current by an electric field has been experimentally observed in superconducting W-C nanostructures for the first time. The single-step Ga$^+$ FIBID procedure used to fabricate these nanodevices does not require the usage of resists, and presents great flexibility in the way nanostructures are patterned, providing with great freedom in pattern design and realization. As such, the experimental finding of a significant field-induced critical current modulation in W-C settles the FIBID technique as relevant and convenient for the nanofabrication framework of S-FET nanodevices.

Quantitatively, results retrieved in the W-C nanostructures are comparable to those obtained in similar vanadium-based nanodevices^6^, with a remarkably low value of the critical gate voltage, required to fully quench the superconducting state, of 3 V at a reduced temperature $T/T_c=0.421$. This gate voltage value is comparable to those used in MOSFET technology, and highly competitive in comparison to those reported in other superconducting materials.

A theoretical model based on the Ginzburg–Landau theory is proposed here, appropriately explaining the experimental data. Accounting for the effect of the squeezing of the supercurrent by the electric field, it adds further insight to the puzzling origin of the phenomenon, which has been investigated in different geometries and other materials, and is subjected to controversy at the present time^7,9,16,19,20^.

Nanoelectronics based on the electric field-induced modulation of the superconducting state are bound to see an inspiring development in the coming years. Functional nanodevices built upon this principle, such as gate-controlled NbN nanowires operating with coherent quantum phase slips^44^, and Ti and V rectifiers^15^ have already been realized. The scope of applications in this framework is wide, with the possibility of fabricating qubits, Josepshon junctions, logic gates, and single-photon detectors.
